# Prediction of Clinical Transporter‐Mediated Drug–Drug Interactions via Comeasurement of Pitavastatin and Eltrombopag in Human Hepatocyte Models

**DOI:** 10.1002/psp4.12505

**Published:** 2020-04-23

**Authors:** Simon J. Carter, Bhavik Chouhan, Pradeep Sharma, Michael J. Chappell

**Affiliations:** ^1^ Biomedical and Biological Systems Laboratory School of Engineering University of Warwick Coventry UK; ^2^ Functional & Mechanistic Safety Clinical Pharmacology & Safety Sciences R&D AstraZeneca R&D Gothenburg Sweden; ^3^ Clinical Pharmacology & Quantitative Pharmacology Clinical Pharmacology & Safety Sciences R&D, AstraZeneca R&D Cambridge UK

## Abstract

A structurally identifiable micro‐rate constant mechanistic model was used to describe the interaction between pitavastatin and eltrombopag, with improved goodness‐of‐fit values through comeasurement of pitavastatin and eltrombopag. Transporter association and dissociation rate constants and passive rates out of the cell were similar between pitavastatin and eltrombopag. Translocation into the cell through transporter‐mediated uptake was six times greater for pitavastatin, leading to pronounced inhibition of pitavastatin uptake by eltrombopag. The passive rate into the cell was 91 times smaller for pitavastatin compared with eltrombopag. A semimechanistic physiologically‐based pharmacokinetic (PBPK) model was developed to evaluate the potential for clinical drug–drug interactions (DDIs). The PBPK model predicted a twofold increase in the pitavastatin peak blood concentration and area under the concentration‐time curve in the presence of eltrombopag in simulated healthy volunteers. The use of structural identifiability supporting experimental design combined with robust micro‐rate constant parameter estimates and a semimechanistic PBPK model gave more informed predictions of transporter‐mediated DDIs.

**Study Highlights**

WHAT IS THE CURRENT KNOWLEDGE ON THE TOPIC?

☑ Currently, most *in vitro* models are not guided by structural identifiability analysis, relying on substrate‐only measurements to evaluate transporter inhibition and subsequent drug–drug interaction (DDI) predictions.

WHAT QUESTION DID THIS STUDY ADDRESS?

☑ Whether using micro‐rate constants compared with macro‐rate constants to describe the comeasurement of uptake of substrate (pitavastatin) and inhibitor (eltrombopag) can improve model fits through structurally identifiable mechanistic models, and the potential for transporter DDIs in the clinic.

WHAT DOES THIS STUDY ADD TO OUR KNOWLEDGE?

☑ The comeasurement of pitavastatin and eltrombopag guided structurally identifiability analysis and improved model fits through micro‐rate constants compared with macro‐rate constants in human hepatocytes, with additional information provided regarding transporter binding. The parameter estimates were then scaled to a semimechanistic physiologically‐based pharmacokinetics (PBPK) model to predict potential clinical interactions.

HOW MIGHT THIS CHANGE DRUG DISCOVERY, DEVELOPMENT, AND/OR THERAPEUTICS?

☑ Micro‐rate constants provide a more dynamic view of binding and translocation, furthering the understanding of transporter pharmacology compared with macro‐rate constants, which can be used in the development of PBPK models and thereby decrease the risk for clinical transporter‐mediated DDIs.

Pitavastatin, one of the family of 3‐hydroxy‐3‐methyl‐glutaryl‐CoA (HMG‐CoA) reductase inhibitors used to manage hypercholesterolemia, has been determined *in vitro* to be a substrate of organic anion transporting polypeptide (OATP)1B1 and OATP1B3 (fraction transported of 0.78) and of the efflux transporters breast cancer resistance protein (BCRP) and multidrug resistance associated protein (MRP) 2.^1^, ^2^ Pitavastatin is more sensitive to transporter inhibition than rosuvastatin *in vitro* as well as in healthy volunteers^2^ and is therefore a good candidate for evaluating transporter‐mediated drug–drug interactions (TrDDIs). Elimination of pitavastatin through metabolism and urinary excretion is relatively small compared with the biliary elimination of pitavastatin (53%).^3^

Eltrombopag is a thrombopoietin agonist used in the management of thrombocytopenic purpura, and the dose is individualized based on the platelet count to prevent excessive clotting or a lack of effect.^4^ It is highly protein bound, and the adsorption to plasma proteins was included to obtain an inhibition concentration at half of the maximum inhibition (IC_50_) value that explained the inhibition of rosuvastatin.^5^ *In vitro* studies found eltrombopag to be a substrate of OATP1B1, OATP2B1, organic cation transporter 1 (OCT1), and BCRP and is also able to inhibit probe substrates for each transporter.^5^, ^6^ The uptake by OATP1B1 is disputed perhaps because of the large amount of nonspecific binding to plastic.^4^, ^6^

Structural identifiability analysis considers the uniqueness of the unknown model parameters from the proposed input–output model structure corresponding to the proposed data collection used for parameter estimation.^7^, ^8^, ^9^, ^10^ This is an important, but often overlooked, theoretical prerequisite to experiment design and parameter estimation because estimates for unidentifiable parameters are effectively meaningless. It is therefore important from a systems pharmacology approach to evaluate, assuming noise‐free data, whether the proposed mathematical model is at least structurally locally identifiable.^7^, ^8^, ^9^, ^10^ Evaluation of TrDDIs *in vitro* is normally conducted without the comeasurement of both substrate and inhibitor in the same sample, assuming that the inhibitor is equal in the medium and cellular compartments using Michaelis‐Menten kinetics. This can lead to the structural unidentifiability of the model, affecting the robustness of estimated parameters, upon which critical decisions may be based.^7^ However, unlike the use of Michaelis‐Menten kinetics, the structural identifiability of micro‐rate constant mechanistic models are unaffected by this assumption.^11^, ^12^

Clinical drug–drug interactions (DDIs) have been observed between eltrombopag and rosuvastatin (as a perpetrator^13^) and lopinavir–ritonavir (as a victim^14^). The main cause of the interaction of eltrombopag with rosuvastatin *in vitro* was the result of BCRP inhibition, with minimal inhibition of OATP1B1.^5^, ^15^ This was confirmed in a semimechanistic physiologically‐based pharmacokinetic (PBPK) model comprising the gastrointestinal tract, liver extracellular space, liver, and a central compartment to adequately describe the interaction between rosuvastatin and eltrombopag.^5^

The aim of this article is to describe the TrDDI between pitavastatin (substrate) and eltrombopag (inhibitor) using an *in vitro* micro‐rate constant mechanistic modeling approach. The micro‐rate constants were used to describe the dynamic transporter interactions following the comeasurement of both in cryopreserved human hepatocytes. The obtained parameters from the *in vitro* experiment were then scaled to a semimechanistic PBPK model to evaluate the interaction between pitavastatin and eltrombopag in simulated healthy volunteers and compared with a static model used to predict clinical interactions (*R *value, US Food and Drug Administration (FDA) guidance document^16^).

## Methods

### Chemicals

Eltrombopag and pitavastatin calcium were obtained from Toronto Research Chemicals (Toronto, Ontario, Canada), cesium chloride (C3032), mineral oil (69794, density 0.872 g/L), oil red O (O0625), dimethyl sulfoxide (DMSO, ≥99.5%), formic acid (99%) and 5,5‐diethyl‐1,3‐diphenyl‐2‐iminobarbituric acid (S518891) were obtained from Sigma‐Aldrich (Stockholm, Sweden). Acetonitrile, methanol, Leibovitz L15 (21083027), and silicone oil (15445005, density 1.08 g/L) were of analytical or cell culture grade and obtained from Thermo‐Fisher Scientific Inc. (Gothenburg, Sweden).

### Use of hepatocytes

Human hepatocytes were obtained from BioIVT (Brussels, Belgium; lot number: LYB; 10 donor LiverPool (8 Caucasians, 1 African American, and 1 Hispanic) and thawed according to supplier guidelines in Leibovitz L15 medium. Hepatocytes were kept on ice prior to use and were used within 3 hours of defrosting.

### Incubations

Hepatocytes (viability 84–87%) were preincubated in Leibovitz L15 medium at 1.5 × 10^6^ cells for 15 minutes at 37ºC with either 0.1% DMSO or 45 nmol/mL eltrombopag. Incubations were started by the addition of pitavastatin (0.3–100 nmol/mL, 1 × 10^6^ cells, 0.35% DMSO, 30 nmol/mL eltrombopag final), and 100 µL samples taken at 0.25–30 minutes. Cells were separated using an oil spin method similar to the literature.^11^, ^17^ After separation, the tubes were frozen on dry ice and the bottom layer was cut off. Pitavastatin and eltrombopag were extracted in a stop solution prior to ultra‐performance liquid chromatography–high‐resolution mass spectrometry analysis (see the **Supplemental Material** for full experimental and sample extraction methods).

### Data analysis

The bottom layer concentrations of pitavastatin and eltrombopag were converted to a cellular concentration using a cellular volume (*V*_cell_) of 3 µL/1 × 10^6^ human hepatocytes:^18^

(1) $$\left[\text{cell}\right]\left(\text{nmol}/\text{mL}\right)=\left(\frac{\left[\text{Bottom layer}\right]\times 15.3\;\mu L}{V_{\text{cell}}}\right)/100$$

where *V*_cell_ /1 × 10^5^ cells was 0.3 µL, and the volume of the bottom layer of cesium chloride was 15 µL. A dilution factor of 100 was used to scale from pmol/mL/1 × 10^5^ cells to nmol/mL/1 × 10^6^ cells.

### Structural identifiability analysis

An efficient computerized method to determine the structural local identifiability uses the IdentifiabilityAnalysis package^9^, ^10^ in Wolfram Mathematica 11.3 (Wolfram Research Inc., Champaign, IL). Given a set of ordinary differential equations (ODEs) with an unknown parameter vector and known input and a set of measurable cellular observations (see **Supplemental Material**), this method gives a Boolean answer to the structural identifiability problem, including the list of any unidentifiable parameters.

### Mechanistic modeling in hepatocytes

Transporter kinetics are normally evaluated using Michaelis‐Menten kinetics as opposed to through the use of more dynamic micro‐rate constants^11^ assuming that:^19^, ^20^

1. Association of substrate to transporter (*k*_a.X_) is very rapid compared with dissociation (*k*_d.X_) and thus at equilibrium within a short timeframe
2. The amount of substrate at the transporter is much greater than the total amount of transporter (*T*_o_)
3. The translocation of substrate from transporter into the cell (*k*_t.X_) is the rate‐limiting step in the transporter‐mediated uptake of the substrate

If these assumptions hold, then the Michaelis‐Menten equation can be derived from the micro‐rate constant models (models 1 and 2, **Table** **1**), defined here as macro‐rate constant models (models 3 and 4, **Table** **1**, ^19^, ^20^). To check the validity of these assumptions, the micro‐rate constant and macro‐rate constant models with and without the comeasurement of eltrombopag were evaluated both for competitive (models 1 and 3, respectively; **Table** **1**) and noncompetitive inhibition (models 2 and 4, respectively; **Table** **1**).

**Table 1:** Structural identifiability results and goodness of fit values for all tested mechanistic models

Model	Inhibition type	Measured analytes	Structural identifiability (no. of parameters to be identifiable)	BIC (wBIC)	% RMSRE (ind pop total)
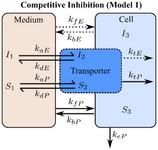	**Competitive**	**Pitavastatin and eltrombopag**	**SI**	**2652 (1)**	**7 + 34* = *41**
	Competitive	Pitavastatin	SI	1096 (1)	9 + 38* = *47
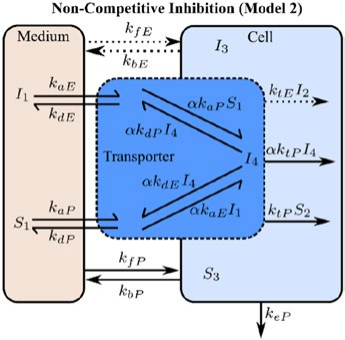	Noncompetitive	Pitavastatin and eltrombopag	SI	2681 (0)	7 + 31* = *38
	Noncompetitive	Pitavastatin	SI	1117 (0)	9 + 39* = *48
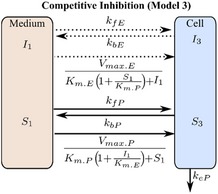	Competitive	Pitavastatin and eltrombopag	SI	2871 (0)	13 + 34* = *47
	Competitive	Pitavastatin	U (1: *K*_m.up.P_ or *K*_I.up.E_)	1386 (0)	16 + 42* = *58
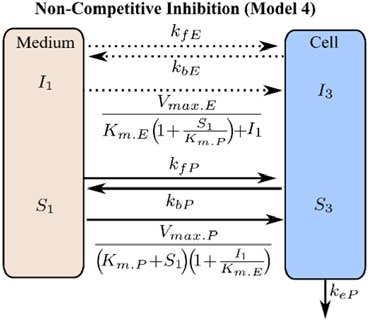	Noncompetitive	Pitavastatin and eltrombopag	SI	2877 (0)	13 + 36* = *49
	Noncompetitive	Pitavastatin	U (1: *V*_max.up.P_ or *K*_I.up.E_)	1378 (0)	16 + 46* = *62

Dotted arrows* =*comeasurement of eltrombopag. Bold font indicates the best‐fitting model based on the percentage of RMSRE, BIC, and wBIC within the same number of timepoints.

BIC, Bayesian information criterion; ind, individual estimate; *K*_I.up.E_, eltrombopag uptake inhibition constant; *K*_m.up.P_, pitavastatin amount at half of the maximum uptake velocity (*V*_max.up.P_); pop, population estimate; RMSRE, sum of the relative mean square root error; SI, structurally (locally) identifiable; U, unidentifiable; wBIC, weighted Bayesian information criterion.

The final chosen mechanistic model (model 1 with the comeasurement of pitavastatin and eltrombopag; **Table** **1**) was based on the outcome of the structural identifiability analysis and goodness‐of‐fit statistics (Bayesian information criterion, and the weighted Bayesian information criterion,^21^ and the sum of the relative mean square root error (percentage RMSRE); see **Supplemental Material**). Model 1 was described using a set of ODEs representing the following: the amount of pitavastatin (*X = *substrate (*S*) or pitavastatin (*P*)) and eltrombopag (*X = *inhibitor (*I*) or eltrombopag (*E*)) in the medium (*X*_1_), the amount bound to transporter (*X*_2_), and the intracellular amount (*X*_3_). Passive rate constants for movement into and out of the cell (*k*_f.X_ and *k*_bX_, respectively) as well as uptake transporter‐mediated rate constants for association to the transporter (*k*_aX_) and dissociation (*k*_dX_) and translocation (*k*_tX_) from the transporter into the medium and cell, respectively. Metabolism of pitavastatin in the cell was through an elimination rate constant (*k*_e_). The sets of ODEs for all the models (models 1–4; **Table** **1**) tested for structural identifiability and used for parameter estimation are included in the **Supplemental Material**.

### Parameter estimation

Parameter estimation was conducted within Monolix 2018 R2 (Lixoft, Antony, France) for each concentration and experiment as an individual data set, where to ensure positivity, a log‐normal distribution was assumed for each parameter, with a proportional residual error model for the observations. Because of the large number of parameters to be estimated in the combined pitavastatin and eltrombopag mechanistic model, the initial estimates for pitavastatin and eltrombopag each were obtained for the micro‐rate constant models (no macro‐rate constant estimates could be obtained for eltrombopag alone for transporter‐mediated uptake).

### Clinical TrDDI assessment

*In vitro* evaluation of the potential for clinical TrDDIs are important in drug development to decrease the risk of adverse events and improve patient quality of life. The FDA in their guidance document for industry^16^ described the use of the static area under the concentration‐time curve (AUC) difference in the presence and absence of inhibitor (the *R *value^1^) used to assess the potential for clinical TrDDIs:

(2) $$R\;\text{value}=1+\frac{f_{u\text{.pl}}I_{\text{in}\text{.max}}}{K_{I}}$$

where *f*_u.pl_ is the fraction unbound in the plasma (set to 0.01 for eltrombopag, the minimum value proposed in the FDA draft guidance^16^ when plasma protein binding ≥ 0.99), *K_I_* is obtained from the micro‐rate constant model (through (eltrombopag amount at half of the maximum uptake velocity (*V*_max.up.E_) (*K*_m.up.E_)), Eq. 12 in **Table** **2**) and:

(3) $$I_{\text{in}\text{.max}}\left(\text{nmol/mL}\right)=\frac{\left(C_{\max+}\left(\frac{\left(F_{a}F_{g}K_{a}\text{Dose}\right)}{Q_{H}}\right)\right)}{R_{b}}$$

**Table 2:** Parameter estimates and scaled estimate physiological values

Model 1: Micro‐rate constant parameter estimates		
Parameter	Estimates for pitavastatin	Estimates for eltrombopag
*k*_aX_ (/nmol/min)	0.17 (0.14–0.25)	0.26 (0.23–0.31)
*k*_dX_ (/min)	2.2 (1.97–2.37)	1.57 (1.42–2)
*k*_tX_ (/min)	1.65 (1.57–1.74)	0.27 (0.24–0.32)
*k*_eP_ (/min)	0.22 (0.2–0.24)	NA
*T*_o_ (nmol)	0.18 (0.11−0.37)	
*k*_fX_ (/min)	5.5 × 10^−4^ (4.6 × 10^−4^–6.1 × 10^−4^)	0.05 (0.04–0.06)
*k*_bX_ (/min)	0.21 (0.18–0.22)	0.43 (0.35–0.65)

PBPK model values (equation or references): drug specific			
*k*_ge_ (/min)		0.1 [^1^]	0.1 [^1^]
*K*_a.X_ (/min)	$\left(\frac{1}{{\text{MRT}}_{\text{PO}}-{\text{MRT}}_{\text{IV}}}/60\right)/k_{\text{ge}}$ (5)	0.07	11^a^
*V*_max.up..X_ (ng/min/liver)	$T_{o}k_{\text{tX}}{\text{MW}}_{X}\cdot \text{HPGL}\cdot \text{LWT}$ (6)	3.0 × 10^7^	5.1 × 10^6^
*K*_m.up.X_ or *K*_I.up.X_ (ng/mL)	$\left(\frac{k_{\text{dX}}+k_{\text{tX}}}{k_{\text{aX}}V_{\text{med}}}\right){\text{MW}}_{X}$ (7)	9,525	3,138
*P*_dif.X_ (mL/min/liver)	$\left(k_{\text{fX}}V_{\text{med}}\right)\cdot \text{HPGL}\cdot \text{LWT}$ (8)	118	1.2 × 10^5^
*P*_def.X_ (mL/min/liver)	$\left(k_{\text{bX}}V_{\text{cell}}\right)\cdot \text{HPGL}\cdot \left(\text{LWT}-V_{\text{ext}\text{.H}}\right)$(9)	104	307
CL_met.X_ (mL/min/liver)	${\mathrm{CL}}_{\text{met}\text{.P}}=\left(k_{\text{eP}}V_{\text{cell}}f_{u\text{.L}\text{.P}}\right)\cdot \text{HPGL}\times \text{LWT}\cdot f_{\text{hep}}$ (10) ${\text{CL}}_{\text{met}\text{.E}}={\left(\text{CL}/F\right)}_{\text{pl}\text{.E}}\cdot F_{E}\cdot \left(1-F_{\text{pl}\text{.E}}\right)$ (11)	93	6.8
CL_bi.X_ (mL/min/liver)	${\text{CL}}_{\text{biP}}={\text{CL}}_{\text{pl}\text{.P}}\cdot f_{\text{fecal}\text{.P}}$ (12) ${\text{CL}}_{\text{biE}}\left(\text{mL}/\min\right)={\left(\text{CL}/F\right)}_{\text{pl}\text{.E}}$ (13)	165	13
CL_ur.P_ (mL/min)	${\text{CL}}_{\text{pl}\text{.P}}\cdot f_{\text{ur}\text{.P}}$ (14)	12	0 parent in urine [^25^]
*f*_u.bl.P or pl.E_	$\frac{f_{u\text{.pl}\text{.P}}}{\text{Bl:Pl}}$ (15)	0.009 and [^3^, ^24^]	0.002 → 0.005 [^4^]
*f*_u.L.X_	$\frac{V_{\text{cell}}}{V_{\text{inc}\text{.RED}}/f_{u\text{.inc}}-V_{\text{med}\text{.RED}}-k_{\text{mem}}}$ (16)	0.026	0.4^b^
*V*_c.X_ (*X*_6_, mL)		5820. Total blood volume [^23^]	2940 [^5^]

PBPK model values (reference): physiological values	
*k*_bile_ * = *0.062/min^39^	*V*_L_ (*X*_4_)* = *1570 mL^23^
*V*_ext.H_ * = X*_3_ vol* = *469 mL^40^	*V*_Gabl_ = *X*_5_ vol* = *36 mL^39^
*Q*_H_ * = *hepatic blood flow* = *1320 mL/min^23^	*Q*_K_ = kidney blood flow* = *1170 mL/min^23^

Parameter estimates are the conditional mode of the conditional distribution (minimum‐maximum) for 1 × 10^6^ cells.

CL/*F*_pl.E_,* *estimated CL/F from the plasma for eltrombopag; CL_bi.X_, biliary clearance of X; CL_met.X_, metabolic clearance; CL_pl.P_, pitavastatin plasma clearance;^3^ CL_ur.P_, urinary clearance of pitavastatin; *F*_E_, approximate eltrombopag bioavailability;^5^ *f*_fecal.P_ and *f*_ur.P_,* *fraction of pitavastatin in the feces and urine, respectively;^3^ *f*_hep_,* *fraction of liver that are hepatocytes* = *0.6;^43^ *F*_pl..E_,* *fraction of total eltrombopag in the plasma;^5^ *f*_u.bl.P_,* *pitavastatin fraction unbound in the blood, obtained from the fraction unbound in the plasma (*f*_u.pl.P_ *= *0.004) and the blood:plasma (Bl:Pl) ratio (0.425); *f*_u.L.P_ *, *fraction unbound in the liver^42^ (obtained from the RED device conditions where *V*_inc.RED_ *,*incubation volume* = *1.203 mL; *f*_u.inc_,* *fraction unbound in the incubation* = *0.89; *V*_med.RED_,* *1.2 mL; and *k*_mem_ *= *approximate membrane volume (1% of cell* = *0.00003 mL)); HPGL,* *hepatocytes per gram liver* = *139;^41^ *K*_a.P_,* *first‐order absorption rate constant for pitavastatin calculated using the mean residence time following oral (PO) and i.v. (IV) administration (MRT_P_);^22^ *k*_aX_ *, k*_dX_ *, k*_tX_,* *transporter association, dissociation, and translocation rate, respectively; *k*_bile_, bile flow rate; *k*_eP_,* *pitavastatin elimination rate constant; *k*_fX_ and *k*_bX_,* *passive rate constants into and out of the cell; *k*_ge_, inverse of the gastric emptying time* = *1/10 minutes;^1^ *K*_m.up.X_,* *concentration at half V_max.up.X_; LWT,* *liver weight for a 83 kg human male; MW_X_ , molecular weight of pitavastatin (421.46) or eltrombopag (442); NA,* *not applicable; *P*_dif.X_ and *P*_def.X_,* *passive diffusion into and out of the cell, respectively;^18^ PBPK, physiologically‐based pharmacokinetic; *T_o_*,* *total amount of transporters; *V*_cell_, volume *per* 1 × 10^6^ cells; *V*_C.X_, volume of the central compartment (blood and plasma for pitavastatin and eltrombopag respectively); *V*_ext.H_,* *volume of liver extravascular space; *V*_Gabl_, volume of the gall bladder; *V_L_*,·liver‐specific gravity* = *1695.6 g;^23^ V_max.up.X_,* *maximum uptake velocity; *V*_med_,* *medium volume per 1 × 10^6^ cells* = *1 mL; *X*,* *pitavastatin (*P*) or eltrombopag (*E*).

^a^ Visual fit to t_max_.

^b^ Visual fit to data.

where peak blood concentration (*C*_max_) is the maximum plasma concentration of the inhibitor (nmol/mL), *F*_a_ *F*_g_ is the fraction absorbed multiplied by the intestinal availability (0.5 for eltrombopag^5^), *K*_a_ is the absorption rate constant set to 0.0084/min for eltrombopag,^5^ *Q*_H_ is hepatic blood flow (**Table** **2**), and Bl:Pl is the blood:plasma ratio of 0.78 for eltrombopag.^5^ An *R *value ≥ 1.1 indicates that a TrDDI is likely.^16^

**Figure 1:**
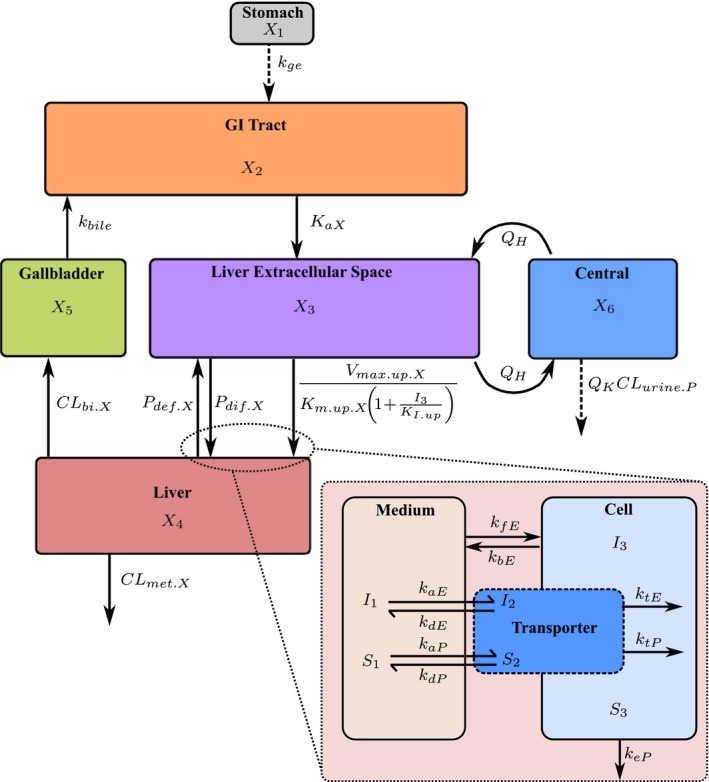
Schematic of the developed semimechanistic physiologically‐based pharmacokinetic model for the concentrations in the liver compartment (*X*_4_) assumed to be involved in the transporter‐mediated drug–drug interactions between pitavastatin and eltrombopag, which is linked to the concentration in the central compartment (*X*_6_) via the concentration in the liver extracellular space (*X*_3_) through *Q*_H_. The dose of pitavastatin or eltrombopag are applied as an amount into the stomach (*X*_1_), which is then transported into the gastrointestinal (GI) tract (*X*_2_) and assumed to be in solution, where *X = *pitavastatin* = P* or eltrombopag* = E*. Drug is absorbed into *X*_3_ where free drug moves into the liver via saturable Michaelis‐Menten kinetics. Uptake is inhibited by the opposing drug in *X*_3_ via the respective uptake concentration at half of the maximum uptake velocity (*K*_m.up.X_) shown as the inhibition constant (*K*_I.up_). Passive movement of drug into and out of the liver is through clearances *P*_dif.X_ and *P*_def.X_, respectively. Both drugs are cleared through biliary excretion (CL_bi.X_) into the gallbladder (*X*_5_), and into *X*_2_ where they can be reabsorbed. Both drugs have metabolic clearance from the liver (CL_met.X_) while pitavastatin is also cleared into the urine (CL_urine.P_) with the kidney blood flow (*Q*_K_). Inset is the *in vitro* mechanistic model for pitavastatin and eltrombopag representing medium (*S*_1_ and *I*_1_), transporter (*S*_2_ and *I*_2_), and within hepatocytes (*S*_3_ and *I*_3_) linked through rate constants obtained during parameter estimation (see **Supplemental Material** for ODEs), which are then scaled accordingly to the whole body (see Eqs. 6–10 in **Table** **2**). *k*_ge_, gastric emptying rate; *k*_bile_, bile flow rate; *K*_a.X_, absorption rate constant of X; Inset parameters: *k*_a.X_, association rate constant of X; *k*_d.X_, dissociation rate constant of X; *k*_t.X_, translocation rate constant of X; *T*_o_, total amount of uptake transporter sites; *k*_f.X_, passive rate constant for movement of X into the cell from the medium; *k*_b.X_, passive rate constant for movement of X into the medium from the cell; *k*_e.P_, pitavastatin elimination rate constant.

A TrDDI was also assessed using a PBPK model, assuming that only the uptake of pitavastatin is inhibited by eltrombopag in humans (and vice versa), with no inhibition of pitavastatin metabolism (see **Figure** **1**). Pitavastatin is metabolized *in vitro* by both UDP‐glucuronosyltransferase (UGT)1A3 and 2B7 and cytochrome P450 (CYP)2C9 and 1B1 and inhibited by eltrombopag (IC_50_ *= *2–20 µM, exception CYP1B1).^4^, ^22^ However, the *in vitro* mechanistic models did not support metabolism, and a simulation including the inhibition of metabolism of pitavastatin did not affect the pitavastatin profile.

The micro‐rate constants for transporter‐mediated uptake and passive uptake were scaled to macro‐rate constants (see **Table** **2**) and then empirically scaled up to the human body (83 kg, healthy adult;^23^ see **Table** **2**). The model was described using ODEs representing the following: The stomach (*X*_1_), the gastrointestinal tract (*X*_2_), the liver extracellular space (*X*_3_), the liver (*X*_4_) with biliary clearance into the gallbladder (*X*_5_) and metabolic clearance as well as a central blood or plasma compartment (*X*_6_; pitavastatin or eltrombopag, respectively), which was linked to *X*_3_ via *Q*_H_. See the **Supplemental Material** for derived ODEs.

The clinical pharmacokinetic data following an oral 1 mg pitavastatin dose in healthy volunteers^2^ were used to validate the pitavastatin PBPK model. As the PBPK model was developed for pitavastatin in the blood, the clinical data were converted to blood (ng/mL) by dividing them by the blood:plasma ratio obtained from the literature (0.425^24^).

Eltrombopag was assumed only to exist in the plasma, because of the high level of binding of eltrombopag to plasma proteins (99.8%^4^), and the volume of distribution reported for a semimechanistic PBPK model (2940 mL^5^). Given the high level of binding, eltrombopag fraction unbound in the plasma values of 0.01,^16^ 0.002,^4^ and 0.005 were considered, with a value of 0.005 giving the best visual fit to the 75 mg clinical data for eltrombopag.^25^ The *F*_a_ *F*_g_ for eltrombopag was reported as 0.5,^5^, ^26^ but was assumed to be 1 in the PBPK model to better visually fit simulations with the clinical eltrombopag data.

The ODE PBPK model (see **Figure** **1** for schematic and **Supplemental Material** for the set of ODEs) was developed using desolve^27^ in R‐studio (v1.1.463, R‐Studio Inc., Boston, MA) running R‐project version 3.6.0^28^ and was similar to that used for eltrombopag and rosuvastatin.^5^ All figures in this article were generated either within Inkscape (0.93, Inkscape.org, Boston, MA) or cowplot, ggplot2,^29^, ^30^ extrafont,^31^ and Cairo.^32^

## Results

### Structural identifiability analysis

The micro‐rate constant mechanistic models tested (see **Supplemental Material**) were at least structurally locally identifiable using the IdentifiabilityAnalysis package,^9^, ^10^ with no parameters unknown, as long as the initial amounts of drug and outputs (measurement of cellular pitavastatin with or without measurement of eltrombopag) were known (see **Table** **1**). If the cellular concentrations of pitavastatin and eltrombopag were both measured as outputs, then the macro‐rate constant mechanistic models tested were at least structurally locally identifiable with no parameters unknown (see **Table** **1**). If only cellular pitavastatin concentration was measured, the macro‐rate constant models were unidentifiable unless one of the parameters was known depending on the inhibition type (see **Table** **1**).

For the PBPK model, assuming known doses of pitavastatin and eltromboag and outputs (measurement of pitavastatin and eltrombopag in the central compartment), the model was at least structurally locally identifiable with no parameters unknown if the following were assumed to be known: Volumes and blood flows and the fraction unbound in the blood or plasma and tissues.

### Mechanistic modeling in hepatocytes

The micro‐rate constant mechanistic model with comeasurement of pitavastatin and eltrombopag and mutual competitive inhibition (**Table** **1**, model 1), had a smaller total RMSRE compared with the measurement of pitavastatin (41% and 47%, respectively; see **Table** **1**). Neither noncompetitive inhibition of pitavastatin uptake nor any of the macro‐rate constant models were supported as the best‐fitting model (see **Table** **1**) and are not discussed further.

**Figure 2:**
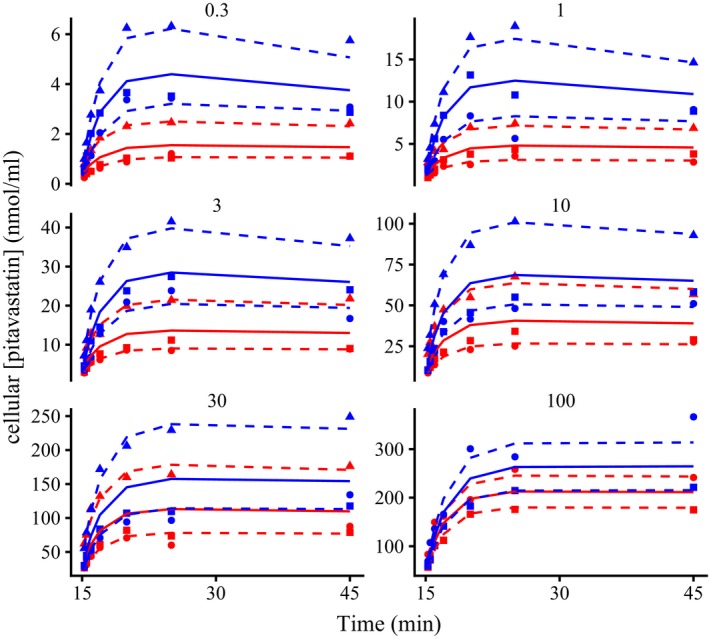
Plots of hepatocyte cell concentration against time over 30 minutes for pitavastatin (0.3–100 nmol, blue, normalized to *t = *15 minutes) with and without 15 minute preincubation with eltrombopag (red). Points are data from three separate experiments. Solid lines are the median pitavastatin individual fits with measurement of eltrombopag. Dashed lines are the maximum and minimum individual fit from Monolix 2018 R2 (Lixoft, Antony, France).

**Figure 3:**
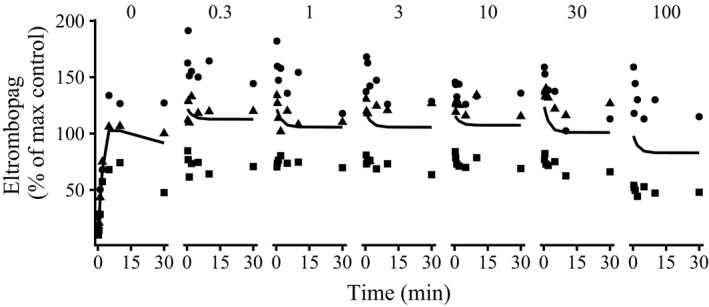
Plots of percentages of the maximum eltrombopag only cellular concentration against time over 30 minutes for eltrombopag in the presence of pitavastatin (0.3–100 nmol) added at *t = *15 minutes (normalized to *t = *0 minutes). Shapes are data from three separate experiments. Solid lines are the median individual fits from the micro‐rate constant model. Subplots are separated by dose of pitavastatin and eltrombopag‐only control (0).

Model 1 (see **Table** **1** and **Figure** **1** inset) visually fit the pitavastatin data well (**Figure** **2**, blue lines; **Table** **1**, individual RMSRE* = *7%), with an initial increase in the cellular pitavastatin concentration at lower incubation concentrations to a maximum followed by a decrease as a result of loss from the cell via metabolism. Pitavastatin uptake was saturated as the incubation concentration increased. In the presence of eltrombopag, the transporter‐mediated uptake of pitavastatin was reduced (**Figure** **2**, red lines), which could be overcome by increasing the pitavastatin incubation concentration until the fits overlapped the pitavastatin‐only data (**Figure** **2**, 10–100 nmol/mL). The mechanistic model was able to visually recover the eltrombopag concentrations in the presence of pitavastatin, with an initial decrease to a new minimum (**Figure** **3**). Because of the variability in the measured cellular eltrombopag concentration across the three experiments (**Figure** **3**, points), the data are displayed after normalization to a 30 nmol/mL incubation of eltrombopag alone (**Figure** **3**, “0”). At higher pitavastatin incubation concentrations (30 and 100 nmol/mL), the percentage of the eltrombopag‐only control was lower than at lower pitavastatin incubation concentrations. The passive rate constant into the cell for pitavastatin (*k*_fP_) was 380 times lower than the passive rate constant out of the cell (*k*_bP_) and 91 times lower than the passive rate constant into the cell for eltrombopag (*k*_fE_) (see **Table** **2**). The passive rate constant out of the cell for eltrombopag (*k*_bE_) was twofold greater than *k*_bP_ (see **Table** **2**). Transporter association (*k*_a.X_) and dissociation (*k*_d.X_) rate constants were similar between pitavastatin and eltrombopag (see **Table** **2**). The difference in transport between the two substrates was in the transporter translocation rate constant (*k*_t.X_) where pitavastatin was sixfold greater than eltrombopag (see **Table** **2**).

For pitavastatin, a *f*_u.inc_ *= *0.9 (relative standard error *= *5%) and gave an estimated *f*_u.l.P_ *= *0.026 (and **Table** **2**) with an analytical recovery of 55%. For eltrombopag, the *f*_u.inc_ *= *0.04 (relative standard error *= *5%) gave an estimated *f*_u.cell_ *= *1.2 × 10^‐5^ and a low analytical recovery of 10%, making the estimate unreliable. This was reflected in the necessary adjustment of fraction unbound in the liver for eltrombopag in the PBPK model (*f*_u.L.E_ *= *0.4; see **Table** **2**).

### PBPK model

**Figure 4:**
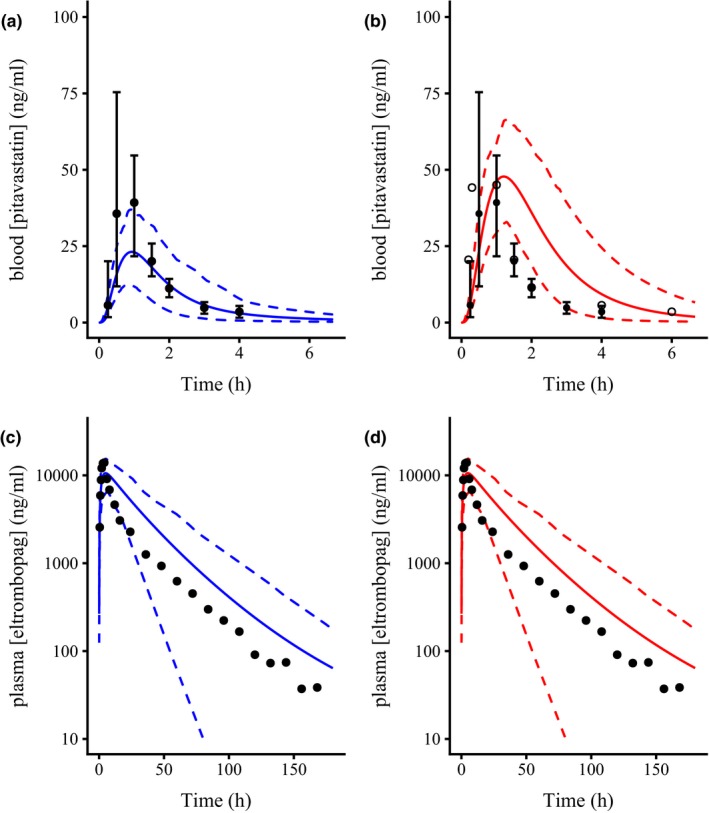
Simulated concentration‐time plots following oral pitavastatin (1 mg) and or eltrombopag (75 mg) administration to healthy volunteers. Semimechanistic physiologically‐based pharmacokinetic (PBPK) model Monte‐Carlo simulation fits (100 subjects) following a 1 mg pitavastatin dose in the absence (**a**, blue, solid lines) and presence (**b**, red, solid lines) of a 75 mg dose of eltrombopag. Semimechanistic PBPK model Monte‐Carlo simulation fits (100 subjects) following a 75 mg eltrombopag dose in the absence (**c**, blue) and presence (**d**, red) of a 1 mg dose of pitavastatin. Filled circles and error bars in **a** and **b** are the pitavastatin clinical data extracted from Prueksaritanont *et al*.^2^ (mean ± standard error of the mean, *n = *8). Open circles in **a** and **b** are the pitavastatin clinical data following a 2 mg dose extracted from the US Food and Drug Administration drug submission document.^22^ Points in **c** and **d** are the eltrombopag clinical data extracted from Deng *et al*.^25^ Dashed lines denote the 95% confidence intervals from the simulation.

Although the PBPK models developed here are based on the drug characteristics and physiologically based simulations of concentration and error, the visual fit for a single 1 mg dose of pitavastatin alone (**Figure** **4a**, blue lines) followed the data reasonably well (**Figure** **4** **a,b**, solid points and error bars, extracted from literature^2^). The C_max_, time to maximum concentration (t_max_), and AUC estimates obtained from the PBPK model for pitavastatin only were similar to the literature^2^ (see **Table** **3**). For eltrombopag alone, the mean simulation following a single 75 mg dose (**Figure** **4c**, blue lines) visually fitted the literature plasma concentration^25^ reasonably well^25^ and closely followed the shape of the plasma concentration vs. time data (**Figure** **4** **c,d**, points, extracted from literature^25^). However, the 5% confidence interval (**Figure** **4** **c,d**, lower dashed lines) increased to large proportions during the eltrombopag elimination phase, likely as a result of a lack of observed elimination of eltrombopag over the hepatocyte uptake time course and a high incubation concentration of eltrombopag. The C_max_ and AUC estimates obtained from the PBPK model following a single 75 mg dose of eltrombopag were similar to the literature,^25^ whereas t_max_ was later (see **Table** **3**).

**Table 3:** Pharmacokinetic parameter estimates for pitavastatin and eltrombopag in blood and plasma, respectively, obtained from the literature and PBPK model simulations

	C_max_	t_max_ (h)	AUC_0−t_ (model) or AUC_0−∞_ (literature)	*R *value	
				Static model	PBPK model
Pitavastatin only (literature)^2^, ^a^, ^a^	38.8 ± 21.9 ng/mL	1 (0.5–1)	68 ± 49 h•ng/mL		
Pitavastatin only (PBPK)	23.1 (12.5–37.0) ng/mL	0.97 (0.82–0.95)	49 (19–95) h•ng/mL		
Pitavastatin (PBPK with eltrombopag)	47.8 (32.9–66.3) ng/mL	1.2 (1.32)	119 (56–212) h•ng/mL	1.06–1.07	2.4 (2.2–3)
Eltrombopag only (literature)^25^	10.9 (8.7–13.6) µg/mL	2.5 (2–4)	145 (101–208) h•µg/mL		
Eltrombopag only (PBPK)	10.6 (6.4–15.4) µg/mL	5.2 (5.2–6.8)	338 (118–628) h•µg/mL		
Eltrombopag (PBPK with pitavastatin)	10.6 (6.4–15.5) µg/mL	5.2 (0.5–6.8)	339 (118–629) h•µg/mL		1

Values are mean ± standard deviations or mean (95% confidence intervals).

AUC, area under the concentration‐time curve; AUC_0−t_, area under the concentration‐time curve to the last timepoint; AUC_0−∞_, area under the concentration‐time curve to infinity; C_max_, peak concentration; PBPK, physiologically‐based pharmacokinetic; t_max_, time to peak concentration.

^a^ Converted to blood concentration. *R *value calculated according to Eq. 216 for static model or the ratio of the AUC in the presence of the inhibitor to the AUC in the absence of the inhibitor for the PBPK model simulation.

Following a 1 mg dose of pitavastatin and a 75 mg dose of eltrombopag, the simulated pitavastatin C_max_ in the blood increased from 23.1 (12.5–37.0) ng/mL to 47.8 (32.9–66.3) ng/mL (**Figure** **4b**, red lines) to above the data for a 2 mg dose of pitavastatin (**Figure** **4b**, open circles, extracted from ref. 22). No effect was seen in the PBPK model fit for eltrombopag in the plasma following a 75 mg dose and a 1 mg dose of pitavastatin (**Figure** **4d**, red lines).

## Discussion

### *In vitro* parameters

If *k*_fP_ is scaled to *P*_dif.P_ (Eq. 7 in **Table** **2**), the value was within the lower range of the literature (*P*_dif.P_ *= *0.55 (0.46–0.61) and 0.4–13 µL/min/1 × 10^6^ cells, respectively^17^, ^33^, ^34^, ^35^). If *k*_b.P_ is also scaled up to *P*_def.P_ (Eq. 8 in **Table** **2**), the value was similar to that obtained with micro‐rate constants by Grandjean *et al*.^11^ (0.62 (0.55‐0.67) and 0.89 (27%) µL/min/1 × 10^6^ cells respectively) and similar to *P*_dif.P_.

The *k*_aP_ estimate obtained for pitavastatin in human hepatocytes over 70 seconds in the literature was more than 40‐fold greater (7.4 (51%)/nmol/min/1 × 10^6^ cells^11^) than the value obtained here (see **Table** **2**), whereas the *k*_dP_ and *k*_tP_ values were of the same order of magnitude (6.3 and 4.3 (85%)/min/1 × 10^6^ cells, respectively,^11^ and see **Table** **2**). The extended time points taken here up to 30 minutes enabled the errors in *k*_dP_ to be determined and reduced the error on *k*_tP_ estimated from the data compared with the literature.^11^ If the transporter rate constants are transformed to *V*_max.up_ and *K*_m.up_ (Eqs. 6 and 7, respectively, in **Table** **2**), then the difference between pitavastatin and eltrombopag can be clearly seen in the *V*_max.up_ *_._* (302 (177–639) and 49 (28–116) pmol/min/1 × 10^6^ cells, respectively), and *K*_m.up_ values (22.1 (16.7–25.5) and 7.1 (7.2–9.6) nmol/mL, respectively). The scaled *V*_max.up.P_ value was similar to the literature (65–354 pmol/min/1 × 10^6^ cells),^11,33,34^ whereas *K*_m.up.P_ was 10‐fold lower than the literature (1.4–2 nmol/mL).^11,33,34^

The similar values for *P*_dif.P_ and *P*_def.P_ confirm experimentally to that assumed in the literature, i.e., passive movement of drug into and out of cell are equal when scaled to a clearance.^17^, ^33^, ^34^, ^35^ However, this assumption only holds when transporter‐mediated uptake dominates over passive uptake and metabolism, calculated from the fraction transported (*F*_T.X_):

(4) $$F_{T\text{.X}}=\frac{{\text{CL}}_{\text{up}.X}}{\left({\text{CL}}_{\text{up}.X}+P_{\text{dif}.X}\right)}$$

where CL_up.X_ *= V*_max.up.X_/*K*_m.up.X_. For pitavastatin *F*_T.P_ *= *0.96 (0.94–0.98), uptake clearance dominates over passive, whereas for eltrombopag, *P*_dif.E_ of 52 (40–64) µL/min/1 × 10^6^ cells dominates compared with a smaller *P*_def.E_ of 1.3 (1.05–1.95) µL/min/1 × 10^6^ cells and *F*_T.E_ *= *0.12 (0.09–0.2). Eltrombopag was reported to show saturable uptake in mouse hepatocytes, attributable to uptake transporters,^5^ but only as an inhibitor in the FDA submission document.^26^ The transporter‐mediated uptake of eltrombopag derived using micro‐rate constants, which was not possible using a macro‐rate constant model, agrees with the findings from mouse hepatocytes,^5^ whereas the *F*_T.E_ value supports the FDA submission document^26^ where passive movement dominates.

### TrDDI risk assessment

The clinical risk of TrDDI according to the *R *value^2^ with eltrombopag as the perpetrator and pitavastatin as the victim was below the FDA cut‐off (1.07 at 75 mg). The FDA label for eltrombopag states that caution should be taken when concomitantly administering eltrombopag with OATP1B1 substrates.^26^ The dose of eltrombopag is closely monitored in the clinic because of its pharmacological effect,^4^ and the *R *values were therefore also calculated with eltrombopag as the victim and pitavastatin as the perpetrator. No effect of pitavastatin on eltrombopag was seen in the experimental data except at a pitavastatin incubation of 30–100 nmol/mL (see **Figure** **3**). This can also be observed with the calculated *R *value below the cut‐offs suggested by the FDA.^16^ This is because of the low dose of pitavastatin given (1–4 mg) in the clinic^2^, ^36^, ^37^ (with an estimated *I*_in.max_ of 0.5–3 nmol/mL) compared with the estimated pitavastatin *K_I_* of 13 (9.5–14) nmol/mL from the micro‐rate constant model.

The large degree of inhibition by eltrombopag on pitavastatin seen in human hepatocytes (**Figure** **2**, red lines) led to the development of a semimechanistic PBPK model (see **Figure** **1**) to evaluate the potential for a clinical TrDDI in a more dynamic environment. The increase in the simulated pitavastatin C_max_ in the presence of eltrombopag from 23.1 (12.5–37.0) ng/mL to 47.8 (32.9–66.5) ng/mL (**Table** **3**) was also seen in the AUC* R *value* = *2.4 (2.2–3) in the presence and absence of eltrombopag (119 (56–212) and 49 (19–95) h•ng/mL, respectively, **Table** **3**), which was greater than the calculated *R *value of 1.06–1.07. The simulated concentration of eltrombopag in the liver extracellular compartment following a 75 mg dose (66,524 ng/mL at 5 minutes) was much larger than the *K*_I.E_ value calculated from the uptake experiment (3,138 ng/mL), making a TrDDI more likely with pitavastatin as the “victim” drug and eltrombopag as the “perpetrator” drug. The inhibition of rosuvastatin by eltrombopag has been attributed to the inhibition of BCRP with little contribution of inhibition of OATP1B1.^5^, ^15^ For the uptake of rosuvastatin to be inhibited to the same extent that pitavastatin was in this study, the calculated IC_50_ had to be 10 times more potent.^5^ Pitavastatin has been shown to be more sensitive to the inhibition of OATP1B1 compared with rosuvastatin with intravenous rifampicin,^2^ and the *K*_I.E_ estimated here for eltrombopag would therefore also be sufficient clinically to inhibit the uptake of pitavastatin into the liver, leading to a clinical TrDDI.^5^, ^15^ The *P*_dif.E_ value (1.2 × 10^5^ mL/min/liver; **Table** **2**) was much greater than the transporter‐mediated clearance for eltrombopag (*V*_max.up.E.WB_/*K*_m.up.E.WB_ *= *1625 mL/min/liver), making a TrDDI unlikely with eltrombopag as the “victim” drug and pitavastatin as the “perpetrator,” seen from the lack of difference in the simulated C_max_, t_max_, and AUC values (see **Table** **3**). The same was concluded for eltrombopag and rosuvastatin.^5^

## Conclusions

By additionally measuring eltrombopag and relying on the large amount of uptake data for pitavastatin in human hepatocytes *in vitro* in the presence and absence of eltrombopag, robust uptake kinetics for eltrombopag and pitavastatin were obtained. The use of a mechanistic modeling approach for parameter estimation, guided by structural identifiability to improve experimental design, shows the advantage of co‐measurement of both substrate and inhibitor. An approach was presented with structural identifiable micro‐rate constant mechanistic models that can aid in promoting systems pharmacology models of transporters and allows the Michaelis‐Menten assumptions for transporters to be formally tested. The improvement in the structural identifiability analysis results and in the model fit seen here using micro‐rate constants compared with macro‐rate constants is in line with previous studies,^11^, ^12^ showing the utility of robust micro‐rate constant mechanistic models in TrDDI analysis in drug development. The developed semimechanistic PBPK model, based on the inhibition of uptake into the liver only, predicted a likely clinical TrDDI between pitavastatin and eltrombopag when compared with use of the static *R *value.

### Future directions

It is recommended to comeasure substrate and inhibitor concentrations in the same sample during *in vitro* TrDDI studies, where a comprehensive mechanistic understanding is required. Further work on developing a robust high throughput of the uptake method (see refs. 12, 38) across more structurally diverse substrates and inhibitors will help increase confidence on the approach presented here using micro‐rate constants.

The robust estimation of the parameters for use in the PBPK model, rather than the use of fixed parameters (with the exception of physiological flows and parameters), and the development of a more micro‐rate constant‐based PBPK model may further the adoption of this approach.

## Funding

S.J.C. was supported by a Collaborative Awards in Science and Engineering studentship from AstraZeneca, Cambridge, UK and Biotechnology and Biological Sciences Research Council Grant 1548253. All experiments and ultra‐performance liquid chromatography–mass spectrometry analyses were carried out at AstraZeneca, Gothenburg, Sweden.

## Conflict of Interest

P.S. and B.C. are employees and shareholders of AstraZeneca, and rosuvastatin is an AstraZeneca drug. All other authors declare no competing interests for this work.

## Author Contributions

S.J.C., B.C., P.S., and M.J.C. wrote the manuscript. S.J.C., B.C., P.S., and M.J.C. designed the research. S.J.C. and B.C. performed the experiments and analyzed the data.

## Supporting information


**Supplemental Material**

**Model Codes**
