# Supplementary material for: Prediction of Clinical Transporter‐Mediated Drug–Drug Interactions via Comeasurement of Pitavastatin and Eltrombopag in Human Hepatocyte Models
Source: CPT Pharmacometrics Syst Pharmacol. 2020 Apr 23;9(4):211–21. doi: 10.1002/psp4.12505 (PMC7179958; doi:10.1002/psp4.12505)
Supplement: Supplementary file 1 — Supplemental Material [file PSP4-9-211-s001.docx]

Authors: Simon J. Carter, Bhavik Chouhan, Pradeep Sharma and Michael J. Chappell

Title: Improved PBPK Prediction of Transporter Mediated Drug-Drug Interactions via Co-measurement of Pitavastatin and Eltrombopag in Human Hepatocyte Models

**Supplemental Data**

***Hepatocyte Incubations.*** 0.4 mL of hepatocytes was diluted 1:1 with Leibovitz either containing DMSO blank (0.1 % for pitavastatin or 0.25 % for eltrombopag alone) or eltrombopag at 90 nmol/mL and pre-incubated at 1.5x10^6^ cells/mL for 15 min at 37 ºC in 7 mL glass scintillation vials (PerkinElmer, Vasby, Sweden) in a shaking water bath (Grant Instruments, Cambridge, UK). Pitavastatin was dissolved and serially diluted in DMSO to give 0.12, 0.4, 1.2, 4, 12 and 40 µmol/mL, and then diluted 400-fold into 37 ºC Leibovitz L15 medium. Eltrombopag was dissolved in DMSO to give 30 µmol/mL, then diluted 333-fold to 90 nmol/mL in Leibovitz L15 medium. Incubations were started by the addition of 0.4 mL of pitavastatin solution at final concentrations of 0.3, 1, 3, 10, 30 and 100 nmol/mL, or by the addition of 0.4 mL eltrombopag at a final concentration of 30 nmol/mL (final DMSO 0.35 % and 1x10^6^ cells/mL hepatocyte suspension). Samples were taken at 0.25, 0.5, 1, 2, 5, 10 and 30 min following the addition of pitavastatin and the cells separated using an oil spin method, similar to Nordell et al. (1) and Grandjean et al. (2). Briefly, 0.5 mL microtubes (12049877, Thermo-Fisher Scientific, Gothenburg, Sweden) layered with 15 µL of 4 % cesium chloride onto which 140 µL of 8:2 silicone oil: mineral oil, with oil red O was added. Samples were taken by transferring 100 µL of hepatocyte suspension into the microtube and spun for 15 s in a Minispin centrifuge (Eppendorf, Horsholm, Denmark), during which time the hepatocytes passed through the oil into the cesium chloride bottom layer. The tubes were then frozen on dry ice and the bottom layer cut off once frozen into a 1 mL deep well plate (260252, Thermo-Fisher Scientific, Gothenburg, Sweden).

***Sample Extraction.*** 50 µL of water suitable for UPLC (MilliQ ELGA water, Merck-Millipore, Solna, Sweden) and 150 µL of stop solution (50:50 MeOH:MeCN, containing 0.8 % formic acid and 4 pmol/mL 5,5-diethyl-1,3-diphenyl-2-iminobarbituric acid as internal standard) were added to the frozen samples of the cut-off tube bottoms. Samples were mixed on a plate shaker for 1 h and then stored at – 80 ºC overnight. Prior to analysis, samples were defrosted and mixed for 30 min before centrifugation of the plates at 4000 g for 20 min at 4 ºC. Samples were transferred to a conical bottomed 96 well plate for analysis (249944, Thermo-Fisher Scientific, Gothenburg, Sweden) and diluted 1:1 with MilliQ ELGA water.

***UPLC-Mass Spectrometry Analysis.*** Samples were analysed by ultra-performance liquid chromatography (UPLC)–high resolution mass spectrometry (HRMS) operated in positive ion mode using an Acquity UPLC I –Class system with column and sample manager and a Xevo TQ-S mass spectrometer (Waters, Sollentuna, Sweden). For HPLC analysis, a Waters Acquity UPLC HSS T3 C18 50 x 2.1mm, 1.8 micron column (Waters, Sollentuna, Sweden) was used with a flow rate of 1mL/min at 40 °C. Mobile phase A was composed of 0.1 % formic acid in MilliQ ELGA water, and mobile phase B was composed of acetonitrile and 0.1% formic acid. The gradient system used was as follows: initially, 0.2 % of mobile phase B was held for 1.3 min followed by a linear gradient to 95 % of mobile phase B from 1.3-1.8 min, and finally 0.2 % of mobile phase B up to 2 min. Injections of 0.3-1 µL were made, depending on incubation dose of pitavastatin. For MS analysis the following settings were used: capillary voltage 0.5 kV; desolvation temperature 600 °C; cone gas flow 150 L/h; nebulizer gas 7.0 bar; collision gas flow 0.15 mL/min. The mass spectrometer was operated in a selected positive ion scanning mode, monitoring for the protonated masses of pitavastatin (422.177>290.153) and eltrombopag (443>182.95). The internal standard was monitored at 336.21>194.99. The UPLC-MS data were acquired, processed and analysed using TargetLynx software (version 4.1, Waters, Sollentuna, Sweden). Eltrombopag and pitavastatin standard curves were linear up to 1000 pmol/mL with R^2^ > 0.96 and a limit of quantitation of 4 pmol/mL and 1 pmol/mL respectively.

***Fraction Unbound in Hepatocytes.*** The estimation of the fraction unbound in hepatocytes at equilibrium can be obtained using multiple techniques: heat inactivated hepatocytes (3); linear regression obtained using the log_D7.4_ (4); parameter estimation (1, 3, 5, 6) or as separate binding rate constants determined experimentally (7). Heat inactivated hepatocytes (3) were used to evaluate the amount of binding across three pitavastatin concentrations (0.3, 10 and 100 nmol/ml) or at 30 nmol/ml eltrombopag overnight using a Rapid Equilibrium Dialysis (RED) device. The RED device works with low volumes across a medium chamber (500 µL, white) and incubation chamber containing the hepatocytes (300 µL, red), and as such has been reported to even out the non-specific binding (NSB) between the membrane and wells, thereby improving recovery (8). The free fraction in the incubation (*f_u.inc_*) is calculated as:

$f_{u.inc}= \frac{[medium chamber] \left( White \right)}{\left[ incubation chamber \right](Red)}$ . (Eq.S1)

The amount of non-specific binding (NSB) to the labware is important to understand the recovery from the RED device experiment to evaluate the reliability of *f_u.inc_*:

$\% recovery=100\times\frac{300\cdot Red+500\cdot White}{300\cdot[incubation]}$ . (Eq.S2)

***Mechanistic Modeling.*** A requirement for the micro-rate constant models is that the amount of free transporters (*T_f_*) available for binding and subsequent uptake of pitavastatin and eltrombopag are known, but this is not normally the case. An estimate for the total amount of transporters (*T_o_*) can be obtained during the mechanistic modeling process (9, 10), and therefore *T_f_* can be replaced using the conservation law during parameter estimation:

$T_{f}=T_{o}-S_{2}-I_{2}$ (Eq.S3)

where *S*_2_ and *I*_2_ are the amounts of substrate (pitavastatin) and inhibitor (eltrombopag) bound to transporter respectively. To improve the readability of the ODEs in Supplemental Table 1, the notation for *T_f_* is used.

As the mode of inhibition of eltrombopag on uptake transporters is not definitively known, but is assumed to be through competitive inhibition (11, 12), both competitive and non-competitive modes of inhibition were evaluated both for micro-rate constant mechanistic models (Supplemental Fig. 1b and c respectively) and macro-rate constant mechanistic models (Michaelis-Menten kinetics, Supplemental Fig. 1e and f respectively). To allow for the simultaneous estimation of both pitavastatin and eltrombopag parameters, compartmental mechanistic models for pitavastatin only were included for the micro-rate constant and macro-rate constant mechanistic models for parameter estimation (Supplemental Fig. 1a and d respectively).

Parameter estimation for all model were conducted within the Monolix suite 2018 R2 (Lixoft, Antony, France) where to ensure positivity, a log-normal distribution was assumed for each parameter in the candidate models, together with a proportional residual error model for the observations. Within the mechanistic modelling processes presented here, it was not known *a priori* whether or not measurement of cellular eltrombopag in addition to measurement of pitavastatin would improve the model fits to the data. Therefore, models with and without measurement of eltrombopag needed to be evaluated, both for competitive and non-competitive inhibition, assuming that both pitavastatin and eltrombopag are substrates of uptake transporters (see Supplemental Fig. 1 and Supplemental Table 1 for tested mechanistic models).

Due to the large number of parameters to be estimated in the combined pitavastatin and eltrombopag mechanistic models, initial estimates for pitavastatin, and eltrombopag only were obtained for the micro-rate constant models (no macro-rate constant estimates could be obtained for eltrombopag alone). In all the datasets, the pitavastatin data at 100 nmol/mL were excluded from one experiment, due to the large concentrations present that adversely affected the total amount of transporters to more than double the rest of dataset (data not shown) and therefore the fits and parameter estimates were also affected. As eltrombopag was pre-incubated in the experimental design at 45 nmol/mL and then diluted to 30 nmol/mL by the addition of pitavastatin, a model for eltrombopag only was used to gain initial parameter estimates. These parameter estimates were then used in a simulation at the 45 nmol/mL using the deSolve package in R (13) to obtain initial conditions for the simultaneous analysis of pitavastatin and eltrombopag, and these were then fitted as free-parameters in the final model.

The final chosen model was based on the structural identifiability analysis results and Bayesian information criterion (BIC) to penalise over-parameterisation within the same set of observations (14) (with or without eltrombopag measurement), as well as the sum of the individual and population prediction of the relative mean square root error (RMSRE):

$\% RMSRE=100\times\sqrt{\frac{1}{n}\sum\left( \frac{{Observed}_{i}-{Predicted}_{i}}{{Observed}_{i}} \right)^{2}}$ (Eq.S4)

where 1/*n* is the inverse of the total number of datapoints multiplied by the sum of the relative squared errors for each datapoint *i*. To evaluate the likelihood of the final model being the best fitting to the data between the BIC values, the weighted BIC (*wBIC*) was calculated according to (15):

${wBIC}_{i}=\frac{exp(-0.5\Delta_{i})}{\sum_{i}^{n} exp(-0.5\Delta_{i})}$ (Eq.S5)

and

$\Delta_{i}={BIC}_{i}- {BIC}_{min}$ (Eq.S6)

where $\Delta_{i}$ is the BIC difference between the current BIC (*BIC_i_*) and the minimum BIC (*BIC_min_*), $exp(-0.5\Delta_{i})$ is the relative likelihood and denominator of Eq. S5 is the sum of all relative likelihoods.


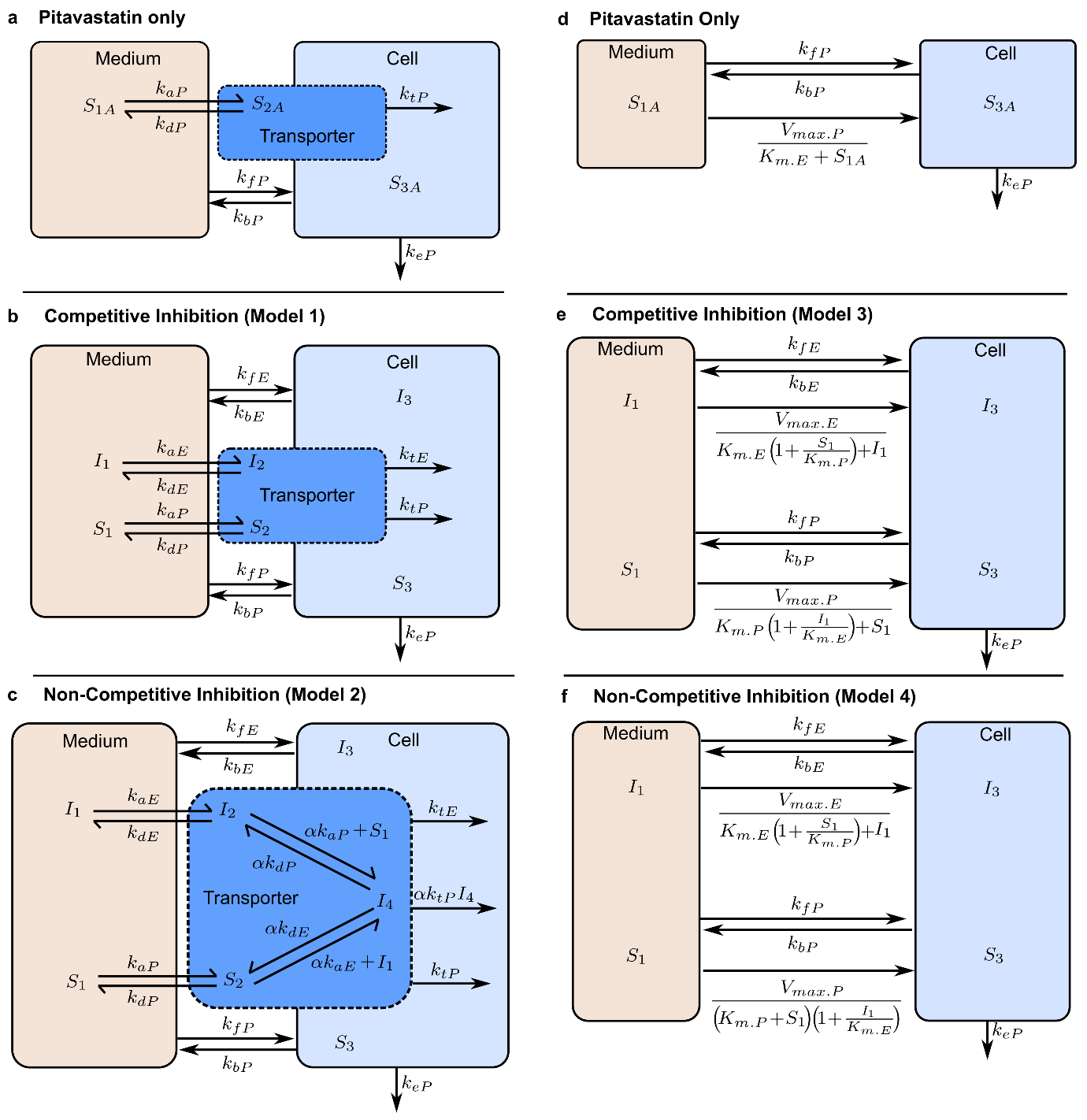


Figure S1: Schematic of the micro-rate constant models (**a-c**, Table S1) consisting of medium, transporter and intracellular compartments, and macro-rate constant models (**d-f**, Table S2) consisting of medium and intracellular compartments. **a** and **d** pitavastatin only used in parameter estimation. Pitavastatin following pre-incubation with eltrombopag with competitive (**b** and **e**) and non-competitive (**c** and **f**) mode of inhibition respectively. *S*_1_*_A_, S*_2_*_A_* and *S*_3_*_A_* are the states for pitavastatin only representing medium, transporter and intracellular amounts. *X* = *S* or *P* = pitavastatin, or *X* = *I* or *E* = eltrombopag. *X*_1_, *X*_2_, and *X*_3_ are the amount in the medium, at the transporter in intracellular respectively. *I*_4_ is the pitavastatin-transporter-eltrombopag complex. All units are /min unless stated *k_fX_* and *k_bX_* are the passive rate constants for movement into and out of the cell respectively, *k_aX_* (/nmol/min), *k_dX_* and *k_tX_* are the transporter association, dissociation and translocation into the cell respectively. α = the dimensionless non-competitive inhibition constant. *V_max.X_* and *K_m.X_* = the maximum uptake velocity (pmol/min) and the amount at half of the *V_max.X_*. *k_e.P_* = pitavastatin elimination rate constant.

Table S1. Sets of derived ODEs for the micro-rate constant mechanistic models (Models 1 and 2, Fig. S1a-c)

| Compartment (nmol) | Competitive Inhibition (**Model 1**, Fig. S1a and b) | Non-Competitive Inhibition (**Model 2**, Fig. S1a and c) |
| --- | --- | --- |
| Medium (*S*_1_*_A_*) | $\frac{{dS}_{1A}}{dt}=-k_{aP}S_{1A}T_{f}-k_{fP}S_{1A}+k_{dP}S_{2A}+k_{bP}S_{3A}$ (Eq.S7) | |
| Transporter (*S*_2_*_A_*) | $\frac{{dS}_{2A}}{dt}= k_{aP}S_{1A}T_{f}-\left( k_{dP}+k_{tP} \right)S_{2A}$ (Eq.S8) | |
| Intracellular (*S*_3_*_A_*) | $\frac{{dS}_{3A}}{dt}= k_{fP}S_{1A}+ k_{tP}S_{2A}-(k_{bA}+k_{eP})S_{3A}$ (Eq.S9) | |
| Medium (*X*_1_) | $\frac{{dS}_{1}}{dt}=-k_{aP}S_{1}T_{f}-k_{fP}S_{1}+k_{dP}S_{2}+k_{bP}S_{3}$ (Eq.S10)  $\frac{{dI}_{1}}{dt}=-k_{aE}I_{1}T_{f}-k_{fE}I_{1}+k_{dE}I_{2}+k_{bE}I_{3}$ (Eq.S11) | $\frac{{dS}_{1}}{dt}= -k_{aP}S_{1}T_{f}-k_{fP}S_{1}+k_{dP}S_{2}+k_{bP}S_{3}-k_{aP}\alpha S_{1}I_{2}+k_{dP}\alpha I_{4}$ (Eq.S18)  $\frac{{dI}_{1}}{dt}= -k_{aE}I_{1}T_{f}-k_{fE}I_{1}+k_{dE}I_{2}+k_{bE}I_{3}-k_{aE}\alpha S_{2}I_{1}+k_{dE}\alpha I_{4}$ (Eq.S19) |
| Transporter (*X*_2_ or *I*_4_) | $\frac{{dS}_{2}}{dt}= k_{aP}S_{1}T_{f}-\left( k_{dP}+k_{tP} \right)S_{2}$ (Eq.S12)  $\frac{{dI}_{2}}{dt}= k_{aE}I_{1}T_{f}-\left( k_{dE}+ k_{tE} \right)I_{2}$ (Eq.S13) | $\frac{{dS}_{2}}{dt}= k_{aP}S_{1}T_{f}-\left( k_{dP}+k_{tP} \right)S_{2}-k_{aE}\alpha S_{2}I_{1}+ k_{dE}\alpha I_{4}$ (Eq.S20)  $\frac{{dI}_{2}}{dt}= k_{aP}I_{1}T_{f}-(k_{dE}+k_{tE})I_{2}-k_{aP}\alpha S_{1}I_{2}+(k_{dP}+k_{tP}){\alpha I}_{4}$ (Eq.S21)  $\frac{{dI}_{4}}{dt}= k_{aE}\alpha S_{2}I_{1}+k_{aP}\alpha S_{1}I_{2}-(k_{dP}+k_{tP}+k_{dE})\alpha I_{4}$ (Eq.S22) |
| Intracellular (*X*_3_) | $\frac{{dS}_{3}}{dt}= k_{fP}S_{1}+ k_{tP}S_{2}-(k_{bA}+k_{eP})S_{3}$ (Eq.S14)  $\frac{{dI}_{3}}{dt}= k_{fE}I_{1}+ k_{tE}I_{2}-k_{bE}I_{3}$ (Eq.S15) | $\frac{{dS}_{3}}{dt}= k_{fP}S_{1}+ k_{tP}S_{2}-(k_{bP}+k_{eP})S_{3}+ k_{tP}\alpha I_{4}$ (Eq.S23)  $\frac{{dI}_{3}}{dt}= k_{fE}I_{1}+ k_{tE}I_{2}-k_{bE}I_{3}$ (Eq.S24) |
| Observations (nmol/ml) | $y_{1}=\frac{1}{V_{cell}}\left( S_{2}+S_{3} \right)$ (Eq.S16)  $y_{2}=\frac{1}{V_{cell}}\left( I_{2}+I_{3} \right)$ (Eq.S17) | $y_{1}=\frac{1}{V_{cell}}\left( S_{2}+S_{3}+I_{4} \right)$ (Eq.S25)  $y_{2}=\frac{1}{V_{cell}}\left( I_{2}+I_{3}+I_{4} \right)$ (Eq.S26) |

*X = S* or *P* = pitavastatin, or *X* = *I* or *E* = eltrombopag, *X*_1_ = amount in the medium. *X*_2_ = amount bound to transporter. *X*_3_ = intracellular amount. *I*_4_ pitavastatin-transporter-eltrombopag complex. Units are /min unless stated. *k_a.X_, k_d.X_* and *k_t.X_* = transporter association (/nmol/min), dissociation and translocation rate constants respectively. *T_f_* = amount of free transporters (nmol), α is the degree to which bound eltrombopag affects the pitavastatin affinity to transporter (dimensionless): α <1 improved pitavastatin affinity, α >1 decreased pitavastatin affinity. *k_f.X_* and *k_b.X_* = passive rate constant into the cell and out of the cell respectively. *k_eP_* = pitavastatin elimination rate constant. *V_cell_* = volume per 1x10^6^ cells.

Table S2. Sets of derived ODEs for the macro-rate constant mechanistic models (Models 3 and 4, Fig. S1d-f)

| Compartment (nmol) | Competitive Inhibition (**Model 3**, Fig. S1d and e) | Non-Competitive Inhibition (**Model 4**, Fig. S1d and f) |
| --- | --- | --- |
| Medium (*S*_1_*_A_*) | $\frac{{dS}_{1A}}{dt}= -k_{fP}S_{1A}-\frac{V_{max.up.P}S_{1A}}{K_{m.up.P}+S_{1A}}+k_{bP}S_{3A}$ (Eq.S27) | |
| Intracellular (*S*_2_*_A_*) | $\frac{{dS}_{3A}}{dt}= k_{fP}S_{1A}+\frac{V_{max.up.P}S_{1A}}{K_{m.up.P}+S_{1A}}-(k_{bP}+k_{eP})S_{3A}$ (Eq.S28) | |
| Medium (*X*_1_) | $\frac{{dS}_{1}}{dt}= -k_{fP}S_{1}-\frac{V_{max.up.P}S_{1}}{K_{m.up.P}\left( 1+\frac{I_{1}}{K_{m.up.E}} \right)+S_{1}}+k_{bP}S_{3}$ (Eq.S29)  $\frac{{dI}_{1}}{dt}= -k_{fE}I_{1}-\frac{V_{max.up.E}I_{1}}{K_{m.up.E}\left( 1+\frac{S_{1}}{K_{m.up.P}} \right)+I_{1}}+k_{bE}I_{3}$ (Eq.S30) | $\frac{{dS}_{1}}{dt}= -k_{fP}S_{1}-\frac{V_{max.up.P}S_{1}}{\left( K_{m.up.P}+S_{1} \right)\left( 1+\frac{I_{1}}{K_{m.up.E}} \right)}+k_{bP}S_{3}$ (Eq.S35)  $\frac{{dI}_{1}}{dt}= -k_{fE}I_{1}-\frac{V_{max.up.E}I_{1}}{K_{m.up.E}\left( 1+\frac{S_{1}}{K_{m.up.P}} \right)+I_{1}}+k_{bE}I_{3}$ (Eq.S36) |
| Intracellular (*X*_3_) | $\frac{{dS}_{3}}{dt}= k_{fP}S_{1}+\frac{V_{max.up.P}S_{1}}{K_{m.up.P}\left( 1+\frac{I_{1}}{K_{m.up.E}} \right)+S_{1}}-(k_{bP}+k_{eP})S_{3}$ (Eq.S31)  $\frac{{dI}_{3}}{dt}= k_{fE}I_{1}+\frac{V_{max.up.E}I_{1}}{K_{m.up.E}\left( 1+\frac{S_{1}}{K_{m.up.P}} \right)+I_{1}}-k_{bE}I_{3}$ (Eq.S32) | $\frac{{dS}_{3}}{dt}= k_{fP}S_{1}+\frac{V_{max.up.P}S_{1}}{\left( K_{m.up.P}+S_{1} \right)\left( 1+\frac{I_{1}}{K_{m.up.E}} \right)}-(k_{bP}+k_{tP})S_{3}$ (Eq.S37)  $\frac{{dI}_{3}}{dt}= k_{fE}I_{1}+\frac{V_{max.up.E}I_{1}}{K_{m.up.E}\left( 1+\frac{S_{1}}{K_{m.up.P}} \right)+I_{1}}-k_{bE}I_{3}$ (Eq.S38) |
| Observations (nmol/ml) | $y_{1}=\frac{1}{V_{cell}}S_{3}$ (Eq.S33)  $y_{2}=\frac{1}{V_{cell}}I_{3}$ (Eq.S34) | $y_{1}=\frac{1}{V_{cell}}S_{3}$ (Eq.S39)  $y_{2}=\frac{1}{V_{cell}}I_{3}$ (Eq.S40) |

*X = S* or *P* = pitavastatin, or *X* = *I* or *E* = eltrombopag, *X*_1_ = amount in the medium and *X*_3_ = intracellular amount. *k_f.X_* and *k_b.X_* = passive rate constant into the cell and out of the cell respectively. *V_max.up.X_* = maximum uptake velocity (pmol/min), *K_m.up.X_* = concentration at which 50 % of the transporters are occupied (nmol). *V_cell_* = volume per 1x10^6^ cells.

Table S3. PBPK model system of ODEs following oral administration of pitavastatin (1 mg) and eltrombopag (75 mg) to a 70 kg healthy human (see main text Fig. 1)

| Compartment | Equation |
| --- | --- |
| Pitavastatin Stomach (*S*_1_, ng) | $\frac{{dS}_{1}}{dt}= -k_{ge}S_{1}$ (Eq.S41) |
| Pitavastatin GI Tract (*S*_2_, ng) | $\frac{{dS}_{2}}{dt}= k_{ge}S_{1}-K_{a.P}S_{2}+k_{bile}S_{5}$ (Eq.S42) |
| Pitavastatin Liver Extracellular Space (*S*_3­_, ng/mL) | $\frac{{dS}_{3}}{dt}=\left( -f_{u.bl.P}S_{3}\left( \frac{V_{max.up.P.WB}}{K_{m.up.P.WB}(1+{I_{3}}/{K_{m.up.E.WB})}+f_{u.bl.P}S_{3}}+P_{dif.P.WB} \right)+Q_{H}f_{u.bl.P}\left( S_{6}-S_{3} \right)+K_{a.P}S_{2}+P_{def.P.WB}f_{u.L.P}S_{4} \right)/V_{ext.H}$ (Eq.S43) |
| Pitavastatin Liver (*S*_4_, ng/mL) | $\frac{{dS}_{4}}{dt}=\left( f_{u.bl.P}S_{3}\left( \frac{V_{max.up.P.WB}}{K_{m.up.P.WB}(1+{I_{3}}/{K_{m.up.E.WB})}+f_{u.bl.P}S_{3}}+P_{dif.P.WB} \right)-f_{u.L.P}S_{4}\left( {CL}_{met.p}+{CL}_{bi.P}+P_{def.P.WB} \right) \right)/V_{L}$ (Eq.S44) |
| Pitavastatin Gallbladder (*S*_5_, ng/mL) | $\frac{{dS}_{5}}{dt}={{CL}_{bi.P}f_{u.L.P}S_{4}}/{V_{GaBl}}-k_{bile}S_{5}$ (Eq.S45) |
| Pitavastatin Blood (*S*_6_, ng/mL) | $\frac{{dS}_{6}}{dt}=\left( Q_{H}f_{u.bl.P}\left( S_{3}-S_{6} \right)-Q_{K}{CL}_{urine.P}S_{6} \right)/V_{c.P}$ (Eq.S46) |
| Eltrombopag GI Tract (*I*_2_, ng) | $\frac{{dI}_{2}}{dt}= -K_{a.E}I_{2}+k_{bile}I_{5}$ (Eq.S47) |
| Eltrombopag Liver Extracellular Space (*I*_3_, ng/mL) | $\frac{{dI}_{3}}{dt}=\left( -f_{u.pl.E}I_{3}\left( \frac{V_{max.up.E.WB}}{K_{m.up.E.WB}(1+{S_{3}}/{K_{m.up.P.WB})}+f_{u.pl.E}I_{3}}+P_{dif.E.WB} \right)+Q_{H}f_{u.pl.E}\left( I_{6}-I_{3} \right)+K_{a.E}I_{2}+P_{def.E.WB}f_{u.L.E}I_{4} \right)/V_{ext.H}$ (Eq.S48) |
| Eltrombopag Liver (*I*_4_, ng/mL) | $\frac{{dI}_{4}}{dt}=\left( f_{u.pl.E}I_{3}\left( \frac{V_{max.up.E.WB}}{K_{m.up.E.WB}(1+{S_{3}}/{K_{m.up.P.WB})}+f_{u.pl.E}I_{3}}+P_{dif.E.WB} \right)-f_{u.L.E}I_{4}\left( {CL}_{met.E}+{CL}_{bi.E}+P_{def.E.WB} \right) \right)/V_{L}$ (Eq.S49) |
| Eltrombopag Gallbladder (*I*_5_, ng/mL) | $\frac{{dI}_{5}}{dt}={{CL}_{bi.E}f_{u.L.E}I_{4}}/{V_{GaBl}}-k_{bile}I_{5}$ (Eq.S50) |
| Eltrombopag Plasma (*I*_6_, ng/mL) | $\frac{{dI}_{6}}{dt}=Q_{H}f_{u.pl.E}(I_{3}-I_{6})/V_{c.E}$ (Eq.S51) |

*X* = *S* = pitavastatin or *I* = eltrombopag, *X*_1-6_ represent stomach, the GI Tract where absorption takes place, the liver extracellular space (volume = *V_ext.H_*), the liver (volume = *V_H_*), the gall bladder (volume = *V_GaBl_*), central compartment (volume = *V_c.X_*). All rate constants are /min unless stated, all clearances and blood flows are mL/min. *k_ge_* = gastric emptying rate, *K_a.X_* = absorption rate constant, *k_bile_* = bile emptying rate constant, *f_u.bl.P_* = pitavatastatin fraction unbound in the blood, *V_max.up.X.WB_* = whole body maximum uptake velocity (ng/min), *K­_m.up.X.WB_* = whole body concentration at 50 % of occupied transporters, *P_dif.X.WB_* and *P_def.X.WB_* = passive clearance into and out of the liver respectively, *Q_H_* = hepatic blood flow, *f_u.L.X_*  = fraction unbound in the liver, *V_ext.H_* = hepatic extravascular space, *CL_met.X_* = metabolic clearance, *CL_bi.X_* = biliary clearance, *V_L_* = liver volume, *V_GaBl_* = volume of the gallbladder, *CL_urine.P_* = pitavastatin urinary clearance, *V_c.P_* = blood volume (mL), *f_u.pl.E_* = eltrombopag fraction unbound in the plasma, *V_c.E_* = plasma volume (mL)

**References:**

1. Nordell P, Winiwarter S, Hilgendorf C. Resolving the distribution-metabolism interplay of eight OATP substrates in the standard clearance assay with suspended human cryopreserved hepatocytes. Molecular pharmaceutics. 2013;10(12):4443-51.

2. Grandjean TR, Chappell MJ, Lench AM, Yates JW, O'Donnell CJ. Experimental and mathematical analysis of in vitro Pitavastatin hepatic uptake across species. Xenobiotica; the fate of foreign compounds in biological systems. 2014;44(11):961-74.

3. Paine SW, Parker AJ, Gardiner P, Webborn PJ, Riley RJ. Prediction of the pharmacokinetics of atorvastatin, cerivastatin, and indomethacin using kinetic models applied to isolated rat hepatocytes. Drug metabolism and disposition: the biological fate of chemicals. 2008;36(7):1365-74.

4. Yabe Y, Galetin A, Houston JB. Kinetic characterization of rat hepatic uptake of 16 actively transported drugs. Drug metabolism and disposition: the biological fate of chemicals. 2011;39(10):1808-14.

5. Poirier A, Lavé T, Portmann R, Brun M-E, Senner F, Kansy M, et al. Design, Data Analysis, and Simulation of in Vitro Drug Transport Kinetic Experiments Using a Mechanistic in Vitro Model. Drug Metabolism and Disposition. 2008;36(12):2434-44.

6. Menochet K, Kenworthy KE, Houston JB, Galetin A. Use of mechanistic modeling to assess interindividual variability and interspecies differences in active uptake in human and rat hepatocytes. Drug metabolism and disposition: the biological fate of chemicals. 2012;40(9):1744-56.

7. Baker M, Parton T. Kinetic determinants of hepatic clearance: plasma protein binding and hepatic uptake. Xenobiotica; the fate of foreign compounds in biological systems. 2007;37(10-11):1110-34.

8. Waters NJ, Jones R, Williams G, Sohal B. Validation of a rapid equilibrium dialysis approach for the measurement of plasma protein binding. Journal of pharmaceutical sciences. 2008;97(10):4586-95.

9. Grandjean TR, Chappell MJ, Yates JW, Evans ND. Structural identifiability analyses of candidate models for in vitro Pitavastatin hepatic uptake. Computer methods and programs in biomedicine. 2014;114(3):e60-9.

10. Jacquez JA. **Nonlinear Compartmental Systems**. Compartmental Analysis in Biology and Medicine, 3ed. Ann Arbor, USA: BioMedware; 1996. p. 92.

11. Takeuchi K, Sugiura T, Matsubara K, Sato R, Shimizu T, Masuo Y, et al. Interaction of novel platelet-increasing agent eltrombopag with rosuvastatin via breast cancer resistance protein in humans. Drug metabolism and disposition: the biological fate of chemicals. 2014;42(4):726-34.

12. Elsby R, Martin P, Surry D, Sharma P, Fenner K. Solitary Inhibition of the Breast Cancer Resistance Protein Efflux Transporter Results in a Clinically Significant Drug-Drug Interaction with Rosuvastatin by Causing up to a 2-Fold Increase in Statin Exposure. Drug metabolism and disposition: the biological fate of chemicals. 2016;44(3):398-408.

13. Soetaert K, Petzoldt T, Woodrow-Setzer R. Solving Differential Equations in R. The R Journal. 2010;2(2):5-15.

14. Aho K, Derryberry D, Peterson T. Model selection for ecologists: the worldviews of AIC and BIC. Ecology. 2014;95(3):631-6.

15. Burnham KPA, D. R. **Information and Likelihood Theory**. Model Selection and Multimodel Inference: A practical Information-Theoretic Approach. 2ed: Springer; 2002. p. 60-80.
