# Supplementary material for: Prediction of Clinical Transporter‐Mediated Drug–Drug Interactions via Comeasurement of Pitavastatin and Eltrombopag in Human Hepatocyte Models
Source: CPT Pharmacometrics Syst Pharmacol. 2020 Apr 23;9(4):211–21. doi: 10.1002/psp4.12505 (PMC7179958; doi:10.1002/psp4.12505)
Supplement: Supplementary file 2 — Model Codes [file PSP4-9-211-s002.zip › Code/Monolix/Monolix Models.pdf]

Authors: Simon J. Carter, Bhavik Chouhan, Pradeep Sharma and Michael J. Chappell

Title: Improved PBPK Prediction of Transporter Mediated Drug-Drug Interactions via Co-measurement of Pitavastatin and Eltrombopag in Human Hepatocyte Models

Monolix Files

Datasets

There are both the dataset files for pitavastatin only (Pita_NoEtm) and pitavastatin with the inclusion of eltrombopag (Pita_Etm_All). Below is a summary of the headers and data:

AMT for pitavastatin and eltrombopag is in nmol.

TIME = time normalised to post eltrombopag incubation, i.e. pitavastatin alone without eltrombopag also begins at t = 15 min.

DV = amount of measured pitavastatin and/or eltrombopag in nmol.

ADM = administration trigger, 1 = eltrombopag dose, 2 = pitavastatin dose.

DVID = observed analyte, 1 = pitavastatin, 2 = eltrombopag.

Models

These correspond to the models described in the main article and ODEs in the Supplemental Data:

Model 1 (micro-rate constant, competitive inhibition) = Pita_Etm_Micro_Model_Comp (observed pitavastatin and eltrombopag) and Pita_NoEtm_Micro_Model_Comp (observed pitavastatin only).

Model 2 (micro-rate constant, non-competitive inhibition of pitavastatin) = Pita_Etm_Micro_Model_NC (observed pitavastatin and eltrombopag). If looking at pitavastatin, remove etm from OUTPUT.

Model 3 (macro-rate constant, competitive inhibition) = Pita_Etm_Macro_Model_Comp (observed pitavastatin and eltrombopag) and Pita_NoEtm_Macro_Model_Comp (observed pitavastatin only)

Model 4 (macro-rate constant, non-competitive inhibition of pitavastatin) = Pita_Etm_Macro_Model_NC (observed pitavastatin and eltrombopag). If looking at pitavastatin, remove etm from OUTPUT.
